# Impact of Residual Inducer on Titratable Expression Systems

**DOI:** 10.1371/journal.pone.0137421

**Published:** 2015-09-08

**Authors:** Taliman Afroz, Michelle L. Luo, Chase L. Beisel

**Affiliations:** Department of Chemical and Biomolecular Engineering, North Carolina State University, Raleigh, North Carolina, United States of America; University of Houston, UNITED STATES

## Abstract

Inducible expression systems are widely employed for the titratable control of gene expression, yet molecules inadvertently present in the growth medium or synthesized by the host cells can alter the response profile of some of these systems. Here, we explored the quantitative impact of these residual inducers on the apparent response properties of inducible systems. Using a simple mathematical model, we found that the presence of residual inducer shrinks the apparent dynamic range and causes the apparent Hill coefficient to converge to one. We also found that activating systems were more sensitive than repressing systems to the presence of residual inducer and the response parameters were most heavily dependent on the original Hill coefficient. Experimental interrogation of common titratable systems based on an L-arabinose inducible promoter or a thiamine pyrophosphate-repressing riboswitch in *Escherichia coli* confirmed the predicted trends. We finally found that residual inducer had a distinct effect on “all-or-none” systems, which exhibited increased sensitivity to the added inducer until becoming fully induced. Our findings indicate that residual inducer or repressor alters the quantitative response properties of titratable systems, impacting their utility for scientific discovery and pathway engineering.

## Introduction

Inducible expression systems have proven to be invaluable tools for probing gene function and optimizing the levels of pathway components. These systems traditionally rely on the addition of a small-molecule inducer that enters the cell via passive or active transport. The inducer then interacts with a signaling cascade or an intracellular sensory regulator, thereby modulating gene expression. Classically, transcriptional sensors have been employed as inducible systems to activate or repress transcription in the presence of the inducer [1,2]. These regulators bind an exogenously added molecule or a metabolic intermediate, altering the binding affinity of the sensor for its DNA operator sites. Separately, riboswitches and other RNA-based devices have become alternative means for the conditional control of gene expression [3–8]. Riboswitches undergo conformational changes when bound to their cognate inducer, resulting in modulation of the transcription or translation of downstream genes [9,10]. In all cases, the applied amount of the exogenous inducer can be varied in order to modulate expression of any regulated genes. The addition of a small molecule thus facilitates simple and finely tuned control of gene expression.

With the widespread use of these systems have come reports of inducers being inadvertently present in the culture medium or being manufactured by the cells—what we term ‘residual inducers.’ For instance, tetracycline in animal feed can be carried over into fetal bovine serum, a standard component of tissue culture medium. As a result, this residual tetracycline impacts inducible expression with the Tet-On system commonly used in eukaryotic cells [11]. Separately, some sugar utilization pathways in bacteria (e.g. the D-galactose and N-acetylglucosamine pathways in *E. coli*) can synthesize their inducing sugar, offering a separate source of inducer if these pathways are employed as titratable systems [12–14]. While these complications might lead some to discard these inducible systems or reformulate the growth medium, other systems may not be sufficiently characterized to identify when residual inducer is even present. In either case, the underlying question is how these sources of inducer impact the apparent relationship between exogenously added inducer and expression levels of regulated genes. Understanding this relationship is particularly important for the fine-tuning of gene expression in quantitative genetic studies as well as the construction of gene circuits that are highly sensitive to component parameters.

Here, we employed mathematical modeling and experimental interrogation of inducible systems in *E. coli* to determine how residual inducer impacts the observed response properties. We found that residual inducer shrank the dynamic range and had varying effects on the sharpness and sensitivity of the response, where these effects principally depended on the value of the original Hill coefficient in the absence of residual inducer. We also observed differences between activating and repressing systems and found that residual inducer had a distinct influence on “all-or-none” systems that exhibit bimodal induction. Overall, our results reveal how residual inducer impacts the quantitative properties of inducible systems, providing insights into how the presence of the inducer can be managed when using these systems for fundamental genetic studies and for pathway optimization.

## Materials and Methods

### Bacterial strains and plasmids

The *E. coli* strains, reporter plasmids, and oligonucleotides used in this work can be respectively found in Tables A, B, and C in S1 File. The *E. coli* strains and the pUA66-ParaB reporter plasmid used for the modified L-arabinose utilization pathway were previously reported in [15]. To construct the pUA66-thiC reporter plasmid, the pUA66 plasmid was purified and linearized with BamHI/XhoI. The plasmid backbone was PCR amplified in two parts using the primers sc101.fwd and sc101.rev as well as the primers pUA66.fwd and pUA66.rev. The *thiC* riboswitch and the first 14 codons of *thiC* was amplified using thiC.fwd and thiC.rev from genomic DNA purified from *E. coli* K-12 substrain MG1655. The thiC.fwd and thiC.rev primers possess a 5' overhang sequence that pair with the ends of the amplified halves of pUA66. The synthetic promoter (BBa_J23119 from the Registry of Standard Biological Parts) was encoded in the 5' overhangs of thiC.fwd and pUA66.rev. Gibson assembly was used to assemble the set of three linear pieces of DNA into a single plasmid [16]. Successful recombinants were verified by PCR and by Sanger sequencing.

### Growth conditions and media

Strains harboring the reporter plasmid were streaked out from freezer stocks into LB plates (10 g/L tryptone, 5 g/L yeast extract, 10 g/L NaCl, 1.5% agar) supplemented with kanamycin and the single colonies were then inoculated in 2 mL of LB (10 g/L tryptone, 5 g/L yeast extract, 10 g/L NaCl) or M9 minimal medium (1X M9 salts, 10 μg/mL thiamine, 2 mM MgSO_4_, 0.1 mM CaCl_2_) supplemented with 0.4% glycerol and 0.2% casamino acids or 0.4% D-glucose and grown overnight at 250 rpm and 37°C. The overnight cultures were then back-diluted into 2 mL of the media containing residual inducer, which was supplemented with varying concentration of the applied inducer. The back-diluted cultures were then grown for 6 h under the same conditions to a final ABS_600_ of ~0.4. The antibiotic kanamycin (0.25 μg/ml) was added when appropriate to select for the pUA66 plasmid.

### Flow cytometry analysis

Flow cytometry analysis was conducted with the Accuri C6 flow cytometer (Becton Dickinson) equipped with CFlow plate sampler, a 488 nm laser, and a 530 ± 15 nm bandpass filter to measure GFP fluorescence in *E. coli* similar to previous work [15]. Cells were diluted 1:100 in 1X PBS before running on the flow cytometer. To eliminate events from cell debris and media components, cells were gated based on forward scatter (FSC-H) and side scatter (SSC-H) using a gate set based on experiments with DRAQ5 dye (Thermo Scientific). A lower cutoff of 12,000 au for FSC-H and 500 au for SSC-H was used. At least 20,000 gated events were collected for each sample. The associated dot plots were generated as described previously [12,15].

### Curve fitting

GFP fluorescence values were calculated by subtracting the mean fluorescence of cells harboring pUA66 (autofluorescence) from the mean fluorescence reading of each sample. The Hill equation was then fit to the resulting GFP fluorescence values using the least-squares approach with the natural log of the measured and predicted mean fluorescence values. For the activating system, The Hill equation is given by Eq 1:

$$\text{y = A}\frac{{\left(\text{c}\right)}^{\text{n}}}{{\text{K}}^{\text{n}}\text{+}{\left(\text{c}\right)}^{\text{n}}}{\text{ + A}}_{\text{0}}$$ (1)

where A, K, and n are fit constants, A_0_ is the fluorescence in the absence of the applied inducer, y is the GFP fluorescence, and c is the concentration of the applied inducer. Of the fit values, n is the Hill coefficient, K is the EC_50_ value, and (A + A_0_)/A_0_ is the dynamic range (δ). For the repressing system, an equivalent approach was used to extract the parameter values based on the Hill equation given in Eq 2:

$$\text{y = A}\frac{{\text{K}}^{\text{n}}}{{\text{K}}^{\text{n}}\text{+}{\left(\text{c}\right)}^{\text{n}}}{\text{ + A}}_{\text{0}}$$ (2)

### Mathematical modeling

The modeling was performed by first calculating the relationship between the concentration of applied inducer (c) and the resulting GFP fluorescence (y) for a given concentration of residual inducer (c_0_) as shown for an activating system (Eq 3) or a repressing system (Eq 4):

$$\text{y = A}\frac{{\left({\text{c + c}}_{\text{0}}\right)}^{\text{n}}}{{\text{K}}^{\eta }\text{+}{\left({\text{c + c}}_{\text{0}}\right)}^{\text{n}}}{\text{ + A}}_{\text{0}}$$ (3)

$$\text{y = A}\frac{{\text{K}}^{\text{n}}}{{\text{K}}^{\text{n}}\text{+}{\left({\text{c + c}}_{\text{0}}\right)}^{\text{n}}}{\text{ + A}}_{\text{0}}$$ (4)

We then fit an adapted Hill equation to the resulting values of x and y for an activating system (Eq 5) or a repressing system (Eq 6):

$$\text{y = A'}\frac{{\left(\text{c}\right)}^{\text{n'}}}{{\left(\text{K'}\right)}^{\text{n'}}\text{+}{\left(\text{c}\right)}^{\text{n'}}}{\text{ + A}}_{\text{0}}\text{'}$$ (5)

$$\text{y = A'}\frac{{\left(\text{K'}\right)}^{\text{n'}}}{{\left(\text{K'}\right)}^{\text{n'}}\text{+}{\left(\text{c}\right)}^{\text{n'}}}{\text{ + A}}_{\text{0}}\text{'}$$ (6)

where A’, A_0_’, K’, and n’ are fit constants. The fitting was performed by calculating A’ and B’ according to the following for an activating system (Eqs 7 and 8) or a repressing system (Eqs 9 and 10) and then determining K’ and n’ using the least-squares approach with the natural log of the actual and predicted values of y:

$${\text{A' = A + A}}_{\text{0}}\text{ - y}\left(\text{0}\right)$$ (7)

$${\text{A}}_{\text{0}}\text{' = y}\left(\text{0}\right)$$ (8)

$$\text{A' = y}\left(\text{0}\right){\text{ - A}}_{\text{0}}$$ (9)

$${\text{A}}_{\text{0}}{\text{' = A}}_{\text{0}}$$ (10)

Of the fit values, n’ is the apparent Hill coefficient, K’ is the apparent EC_50_ value, and (A’ + A_0_’)/A_0_’ is the apparent dynamic range (δ’).

## Results

### Predicted impact of residual inducer on an activating titratable system

**Fig 1:**
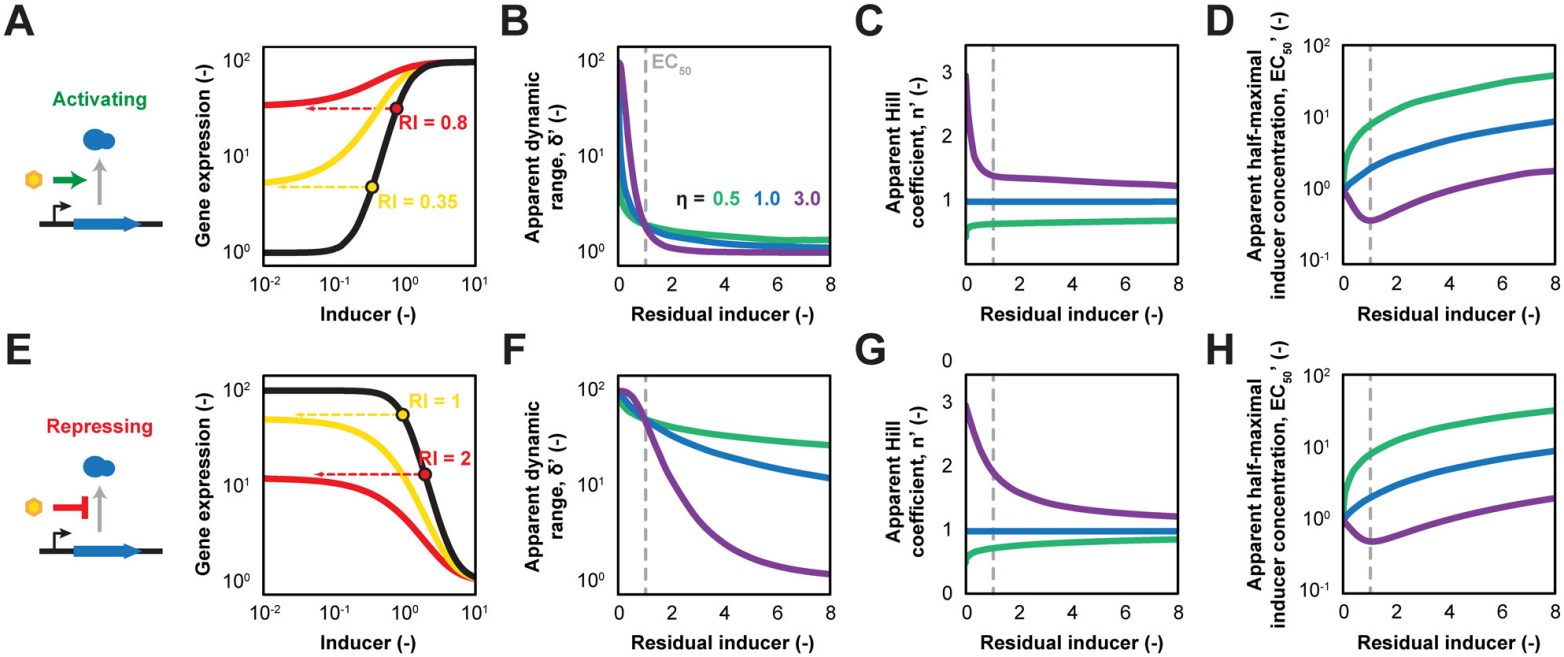
Mathematical modeling of residual inducer on titratable systems. Modeling results for an activating system (**A-D**) and a repressing system (**E-H**) are shown. (**A,E**) Residual inducer (RI) stretches the response curve to lower applied inducer concentrations. The response curve reflects the relationship between the applied inducer concentration and gene induction. (**B,F**) Predicted effect of residual inducer on the apparent dynamic range (δ’). (**C,G**) Predicted effect of residual inducer on the apparent Hill coefficient (n’). (**D,H**) Predicted effect of residual inducer on the apparent half-maximal inducer concentration (EC_50_’). The remaining model parameters were set to A = 100, A_0_ = 1, and EC_50_ = 1.

We first asked how residual inducer would quantitatively impact the apparent response curve of an activating system—the most common type of system employed for inducible control. To capture each response, we employed the Hill equation (Eq 1) that requires three parameters: the Hill coefficient (n) indicative of the sharpness of the response curve, the half-maximal inducer concentration (EC_50_) yielding the average of the maximal and minimal expression levels, and the dynamic range (δ) reflecting the ratio of maximal to minimal gene expression levels. Fig A in S1 File illustrates each parameter and the impact of residual inducer on the corresponding response curve. We modified the Hill Equation (Eq 3) to account for inducer from two sources: applied inducer (c) and residual inducer (c_0_)—whether inadvertently present in the medium or manufactured by the cells. An adapted Hill equation (Eq 5) was then fit to the resulting curves assuming that the inducer added to the medium was the only inducer known to be present in the medium, yielding values for the apparent Hill coefficient (n’), the apparent half-maximal inducer concentration (EC_50_’), and the apparent dynamic range (δ’). This analysis was conducted for varying values of the original Hill coefficient (Fig 1A), EC_50_, and the dynamic range (Fig B in S1 File).

Based on the fit values, we observed trends for the activating system that depended strongly on the Hill coefficient value (n) in the absence of residual inducer (Fig 1A–1D)—what we term the original value. For n > 1, the residual inducer lowered the apparent Hill coefficient (n’) converging on a value of one (Fig 1C). The flatter curve emerges from stretching the response curve from the residual inducer concentration to a value of zero, giving the curve a more graded appearance (Fig A in S1 File). Residual inducer also had a non-monotonic impact on the apparent half-maximal inducer concentration (EC_50_’). While residual inducer would be expected to decrease EC_50_’ due to the response curve stretching, the elevated basal expression increased the expression level associated with the EC_50_’, giving rise to the non-monotonic behavior (Fig 1D). For n < 1, residual inducer caused the apparent Hill coefficient to converge on a value of one (Fig 1C) while solely elevating the apparent half-maximal inducer concentration (Fig 1D). For all values of n, residual inducer greatly impaired the dynamic range (Fig 1B). This effect can be attributed to residual inducer elevating basal expression, leaving only a portion of the response curve for full induction.

We also examined how the trends for the activating system depended on the original EC_50_ and dynamic range (Fig B in S1 File). The responses were similar irrespective of the original EC_50_ or dynamic range values, which was expected based on non-dimensionalization of the Hill equation:

$$\frac{\text{y}}{{\text{A}}_{\text{0}}}\text{ = }\frac{\text{A}}{{\text{A}}_{\text{0}}}\frac{{\left(\frac{\text{c}}{\text{K}}\right)}^{\text{n}}}{\text{1+}{\left(\frac{\text{c}}{\text{K}}\right)}^{\text{n}}}\text{ + 1}$$ (11)

The non-dimensionalization normalizes the values of c and y to the EC_50_ value and the dynamic range (δ = A/A_0_ + 1), respectively, effectively re-scaling the horizontal and vertical axes of the plot. These observations imply that the impact of residual inducer principally depends on the original value of the Hill coefficient.

### Impact of residual inducer for an activating titratable system in *E*. *coli*

To explore these predictions experimentally, we first evaluated the impact of residual inducer on the L-arabinose-inducible P_BAD_ promoter, a common inducible system that originated from *E*. *coli* [17–19]. This promoter is naturally repressed by the sensory regulator AraC through DNA-looping interactions [20]. In the presence of L-arabinose, the AraC dimer undergoes a conformational change and recruits RNA polymerase to initiate transcription. As the P_BAD_ promoter naturally exhibits bimodality in response to L-arabinose [12,21,22], we employed a modified *E*. *coli* strain (MG1655 P_con_-*araE* Δ*araFGH* Δ*araBAD*) which exhibits a unimodal response [15]. The strain was transformed with the low-copy pUA66-ParaB plasmid expressing GFP under the control of the P_BAD_ promoter and grown to mid-log phase in the presence of varying L-arabinose concentrations prior to flow cytometry analysis. We initially compared the effect of adding the residual inducer as part of the overnight culture or during the back-dilution. Both approaches yielded the same response (Fig C in S1 File), prompting us to only add residual inducer as part of the back-dilution in subsequent experiments.

**Fig 2:**
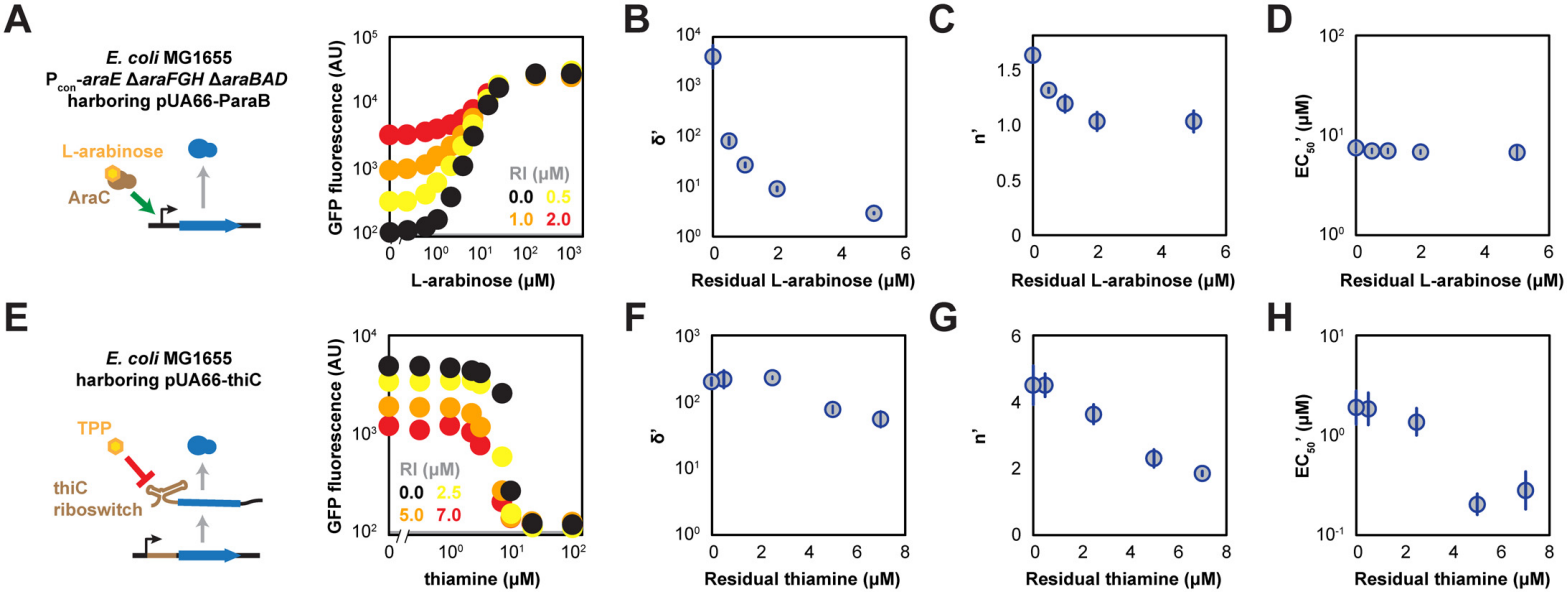
Impact of residual inducer on titratable systems in *E*. *coli*. (**A**) Response curves for *E*. *coli* MG1655 P_con_-*araE* Δ*araE* Δ*araBAD* cells harboring pUA66-paraB. The GFP reporter is activated in the presence of L-arabinose. The concentration of residual L-arabinose (RI) is indicated. The gray bar represents the measured fluorescence of the same *E*. *coli* cells harboring pUA66. Impact of residual L-arabinose on (**B**) the apparent dynamic range (δ’), (**C**) the apparent Hill coefficient (n’), and (**D**) the apparent half-maximal inducer concentration (EC_50_’) for P_con_-*araE* Δ*araE* Δ*araBAD* cells. (**E**) Response curves for *E*. *coli* MG1655 cells harboring pUA66-thiC. The GFP reporter is repressed in the presence of thiamine. The concentration of residual thiamine is indicated. The gray bar represents the measured fluorescence of the same *E*. *coli* cells harboring pUA66. Impact of residual thiamine on (**F**) the apparent dynamic range (δ’), (**G**) the apparent Hill coefficient (n’), and (**H**) the apparent half-maximal inducer concentration (EC_50_’) for MG1655 cells. Values represent the mean and S.E.M. of independent experiments with at least three separate colonies.

We observed trends that followed model predictions when varying the residual concentration of L-arabinose present in the medium (Fig 2A–2D). The apparent dynamic range shrank (from δ’ = 4,000 to δ’ = 3.0) with increasing concentrations of residual L-arabinose (Fig 2B), which approached a value of one at residual L-arabinose concentrations below the original EC_50_ value (7.5 μM, Fig 2D). Second, the apparent response became less steep and approached an apparent Hill coefficient of one (from n’ = 1.6 to n’ = 1.0) before reaching the original EC_50_ (Fig 2C). Finally, the apparent EC_50_ values were roughly independent of residual inducer, which was expected because the residual inducer concentration was below the original EC_50_ value (Fig 2D).

### Predicted impact of residual repressor on a repressing titratable system

We employed a similar approach to predict the impact of residual repressor on repressing systems (Fig 1E). While the activating and repressing systems exhibited similar qualitative trends, we observed quantitative differences that reflect the relative susceptibility of each system to residual inducer or repressor. In particular, the repressing system was less susceptible than the activating system to residual inducer, as higher concentrations of residual repressor were required to shrink the dynamic range (Fig 1F) and alter the slope of the apparent response (Fig 1G). This was particularly apparent for the dynamic range with smaller values of n, which showed a weak dependence on residual repressor (Fig 1F). Aside from the trends for the apparent dynamic range and the apparent Hill coefficient, the trends for the apparent EC_50_ were quantitatively similar between the activating system and the repressing system (Fig 1H). Overall, these results predict that residual inducer or repressor imparts distinct quantitative effects on the perceived response curve that depend on whether the titratable system is activating or repressing.

### Impact of residual repressor for a repressing titratable system in *E*. *coli*

To explore the model predictions, we next evaluated the impact of residual repressor on a repressing system in *E*. *coli*. We selected the thiamine pyrophosphate (TPP)-responsive riboswitch located in the 5’ untranslated region of the *thiC* gene in *E*. *coli*, which represses expression of the *thiCEFSGH* operon in the presence of exogenous thiamine [23]. Naturally, thiamine is imported and enzymatically converted into TPP, which triggers the riboswitch to halt expression of the downstream TPP biosynthetic operon [24]. To employ the *thiC* riboswitch as a repressing system, we constructed a reporter plasmid (pUA66-thiC) in which a synthetic constitutive promoter drives expression of the *thiC* riboswitch and the first 14 codons of the *thiC* gene translationally fused to *gfp* (Fig 2E). *E*. *coli* K-12 MG1655 cells transformed with the pUA66-thiC plasmid were then cultured in varying concentrations of applied thiamine in media already containing residual thiamine.

As shown in Fig 2E–2H, we observed close agreement between the extrapolated experimental values and the model predictions (Fig 1, bottom). The apparent dynamic range and the apparent Hill coefficient both decreased (from δ’ = 200 to δ’ = 55, from n’ = 4.5 to n’ = 1.8) with greater concentrations of residual thiamine (Fig 2F and 2G); as predicted, both parameters continued to decrease even at concentrations of residual thiamine greater than the original EC_50_ value (4.4 μM, Fig 2H). Furthermore, the apparent EC_50_ value exhibited the predicted non-monotonic dependence on residual thiamine, with the trough around the original EC_50_ value (4.4 μM, Fig 2H). These findings further demonstrate the impact of residual repressor on repressing systems, particularly in contrast to activating systems.

The intracellular accumulation of thiamine in *E*. *coli* is known to vary based on the growth conditions and, in particular, the presence of the amino acids [25]. To evaluate how the growth conditions influences the impact of residual thiamine, we repeated the measurements for cells cultured in nutrient-rich LB medium or in M9 minimal medium supplemented with glucose but no casamino acids. LB medium yielded low fluorescence even in the absence of applied thiamine (Fig D in S1 File), which can be attributed to excess quantities of thiamine naturally present in yeast extract. Separately, M9 minimal medium supplemented with glucose but no casamino acids yielded the same qualitative trends as those observed for M9 minimal medium supplemented with glycerol and casamino acids (Fig D in S1 File). These insights provide an additional example in which residual inducer in the medium impacts an inducible system and support the generality of model predictions even under varying growth conditions.

### Impact of residual inducer for an “all-or-none” system in *E*. *coli*

While our modeling and experimental efforts focused on systems that yield unimodal responses, many other inducible systems are known to exhibit bistable or “all-or-none” behaviors [12,26]. This behavior is typified by full or negligible induction in single cells. One prevalent example is the P_BAD_ promoter, which exhibits “all-or-none” behavior in response to exogenous L-arabinose particularly in strains lacking catabolic activity [12,21,22,27]. L-arabinose naturally induces expression of a high-affinity transport system (AraFGH) and low-affinity transport system (AraE) that each import L-arabinose into the cell, resulting in a positive feedback loop that drives maximal activity of the promoter [28].

**Fig 3:**
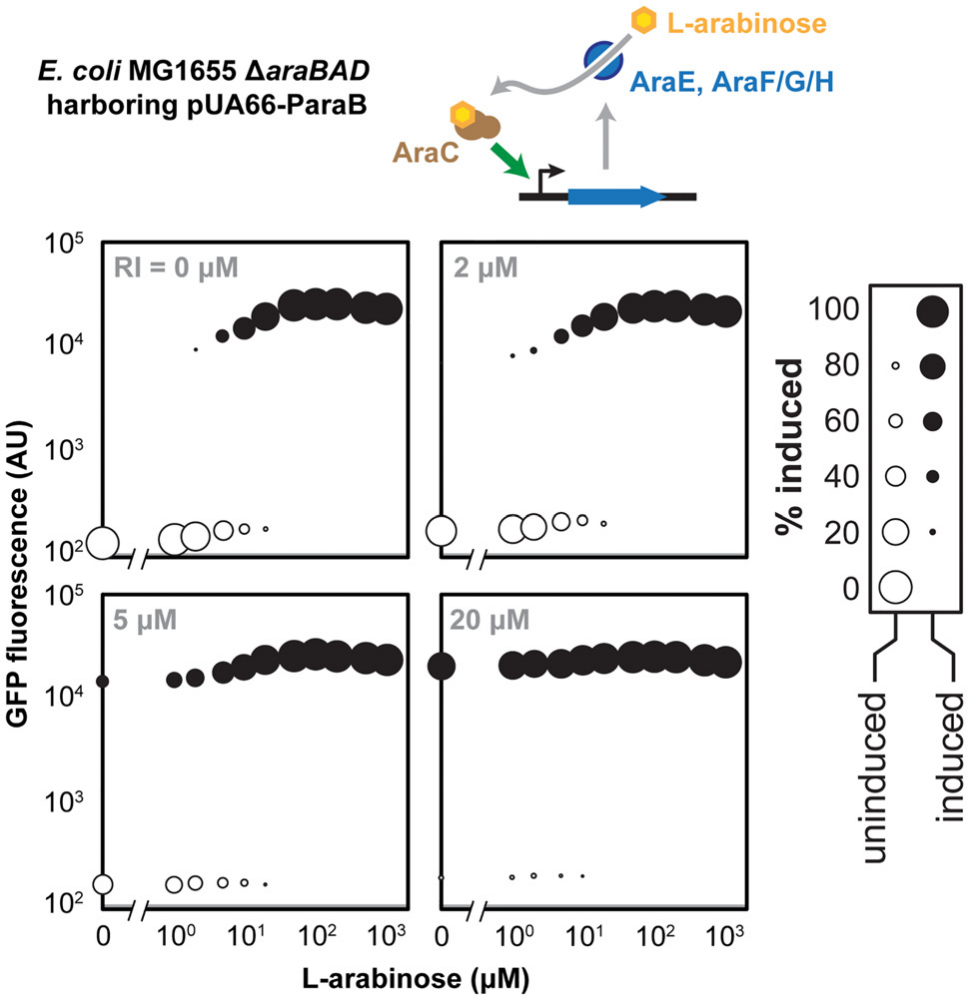
Impact of residual inducer on an “all-or-none” system in *E*. *coli*. (**A**) Dot plots for *E*. *coli* MG1655 Δ*araBAD* cells harboring pUA66-paraB with the indicated concentration of residual L-arabinose. Filled and empty circles represent the induced and uninduced sub-populations, respectively. The size of the dot reflects the proportion of cells in the sub-population, whereas the height of the dot reflects the mean fluorescence of the sub-population. The gray bar represents the measured fluorescence of the same *E*. *coli* cells harboring pUA66. Values are representative of independent experiments starting with at least three separate colonies.

To examine the impact of residual inducer on an “all-or-none” response, we transformed a strain of *E*. *coli* K-12 MG1655 unable to consume L-arabinose (Δ*araBAD*) with the pUA66-ParaB reporter plasmid. The strain was then cultured in medium containing residual L-arabinose, which was supplemented with varying concentrations of additional (applied) L-arabinose. We did not pre-incubate the cells as part of the overnight culture to avoid induction at low L-arabinose concentrations from extended exposure [12]. The resulting bimodal responses were captured as dot plots to communicate the relative abundance and fluorescence of each sub-population (Fig 3).

We first observed that, at lower concentrations, the residual L-arabinose increased the sensitivity of the promoter to the applied L-arabinose (Fig 3). This can be attributed to the residual L-arabinose approaching the concentration in which cells transition from the uninduced state to the induced state. Once the residual L-arabinose reached the switching threshold (~3 μM), a portion of the population was fully induced even in the absence of applied L-arabinose. At higher concentrations of residual L-arabinose, the entire population was fully induced and was no longer responsive to applied L-arabinose. These findings indicate that residual inducer can sensitize an “all-or-none” system to the applied inducer, although excessive amounts of residual inducer can drive the system into the fully induced state.

## Discussion

Using mathematical modeling and reporter assays in *E*. *coli*, we found that the presence of residual inducer reshaped the perceived quantitative properties of a titratable expression system. The inducer generally acted to impair the dynamic range and drive the Hill coefficient toward a value of one. In contrast, the inducer had a complex effect on the EC_50_ value because of the dueling influences of stretching the response curve and increasing the expression level associated with the EC_50_ value. The modeling predictions closely matched the experimental results for the L-arabinose-inducible *araBAD* promoter system and the thiamine-repressible *thiC* riboswitch, suggesting that quantitative effect of residual inducer is independent of the underlying mechanisms of gene induction.

This impact of residual inducer was primarily mathematical in nature, as we effectively rescaled the horizontal axis of the response curve. However, this impact has physical implications when the inducer is inadvertently present in the growth medium or is manufactured by the cells [12–14] by skewing the perceived relationship between inducer concentration and the gene-expression output. The impact of residual inducer is particularly important when the system is not well characterized or the media components are not fully defined. For instance, researchers developing inducible systems in undomesticated microbes may be unaware of residual inducer, whether due to the use of undefined media that may contain trace amounts of inducer, the potential of an endogenous inducible system (e.g. inducible sugar utilization pathway) to also synthesize the inducing molecule, or crosstalk between an imported inducible system and intracellular metabolites. Being able to distinguish between the presence of residual inducer and a system being inherently leaky or poor performing will be important when deciding the overall utility of an inducible system, whether for fundamental genetic studies or for synthetic biology applications.

While residual inducer is normally avoided when working with inducible systems, its presence could be beneficial. For inducible systems with n > 1, the flattening of the response curve yields a more tunable response, allowing finer tuning of expression levels with the applied inducer. The loss of the dynamic range certainly is a detriment, although an intermediate residual inducer concentration may yield a flattened response curve without severely hindering the dynamic range. This would be particularly applicable to systems with large Hill coefficients, such as those associated with cooperativity or stoichiometric binding [29–31]. Note that these potential benefits are limited to systems with n > 1, as systems with n < 1 exhibited a sharper response curve in the presence of residual inducer that would make the system less tunable.

We did observe quantitative differences between activating and repressing systems that impact the influence or potential utility of residual inducer. For instance, repressing systems were less sensitive than activating systems to residual inducer, particularly for n < 1. This translates into background levels of inducers exerting less of an influence on the response properties—an important consideration when choosing between Tet-On (activating system) or Tet-Off (repressing systems) if the medium contains residual tetracycline. The differences that we observed could arise because of distinctions based on the mechanism of regulation, intracellular synthesis of the inducer, and types of layered feedback, and essentiality of the inducer to cell viability. However, our experimental results closely matched modeling predictions even under varying growth conditions, supporting the generality of our findings to a broad range of inducible systems and organisms.

## Supporting Information

S1 File This file contains Tables A-C, Figs A-D, and Supporting References. (PDF)
