# Supplementary material for: Impact of Residual Inducer on Titratable Expression Systems
Source: PLoS One. 2015 Sep 8;10(9):e0137421. doi: 10.1371/journal.pone.0137421 (PMC4562711; doi:10.1371/journal.pone.0137421)
Supplement: S1 File — (PDF) [file pone.0137421.s001.pdf]

SUPPORTING INFORMATION

Impact of residual inducer on titratable expression systems

Taliman Afroz, Michelle L. Luo, and Chase L. Beisel

TABLE OF CONTENTS

Supporting Tables S1 – S3	…	2
Supporting Figures S1 – S4	…	5
Supporting References	…	9

SUPPLEMENTARY TABLES

Supplementary Table S1. Strains used in this study

Name	Genotype	Source	Stock #
MG1655	*Escherichia coli* K-12 substrain MG1655	Gottesman lab (NIH)	CB168
Δ*araBAD*	MG1655 Δ*araBAD::cat*	[1]	CB225
P_{con}-*araE* Δ*araFGH* Δ*araBAD*	MG1655 P_{araE}::[*cat* P_{con}] Δ*araBAD* Δ*araFGH::bla*	[2]	CB328

Supplementary Table S2. Plasmids used in this study.

Name	Description	Source	Stock #
pUA66	Low-copy plasmid (pSC101 ori) with MCS upstream of *gfp*	OpenBiosystems	pCB198
pUA66-ParaB	pUA66 with the *araB* promoter	OpenBiosystems	pCB208
pUA66-thiC	pUA66 with synthetic promoter (BBa_J23119) and portion of the *thiC* riboswitch and the first 14 codons of the *thiC* gene	This study	pCB404

Supplementary Table S3. Oligonucleotides used in this study. Bases shown in red are part of the synthetic promoter (J23110). Bolded bases are the homology regions for recombination into MG1655.

Name	Sequence
del-araBAD.fwd	**TCCATACCCGTTTTTTTGGATGGAGTGAAACG**CATGGTCCATATGAATATCCTCCTTAG
del-araBAD.rev	**GTTTGCTGCATATCCGGTAACTGCGGCGCTAACTGACGGCAG**GTAGGCTGGAGCTGCTT
ParaE.fwd	**TCTGCTGTAAAATTAGGTGGTTAATAATAATCTCAATAATTCA**GTAGGCTGGAGCTGCTT
J23110.rev	**GCTAGCATTGTACCTAGGACTGAGCTAGCCGTAAA**CATATGAATATCCTCCTTAG
ParaE-J23110.rev	**GATAGTAACCATTTTTTCCTGCCAGCAGAGAGTAAGACGCTAGCATTGTACCTAGGACTG**
del-araFGH.fwd	**TCATTCGTTTTTGCCCTACACAAAACGACACTAAAGCTGG**TGATGCCTGGCAGTTCCCTA
del-araFGH.rev	**GACAGTGCGTTTCGCTTTTTGCTTGTAACGGTCGAAGA**TTACCAATGCTTAATCAGTGAG
sc101.fwd	TTGCTTTAGCTAATACACCATAAGCAT
sc101.rev	ATGCTTATGGTGTATTAGCTAAAGCAA
pUA66.fwd	AGTAAAGGAGAAGAACTTTTCACT
pUA66.rev	ATTATACCTAGGACTGAGCTAGCTGTCAAGTGAAGACGAAAGGGCCTCGTG
thiC_fwd	CTAGCTCAGTCCTAGGTATAATGCTAGCAATGCCCCATTTGCGGGGCTAA
thiC_rev	GAAAAGTTCTTCTCCTTTACTGGCGCGTTGTTCGCGGCGGGTCA

SUPPLEMENTARY FIGURES

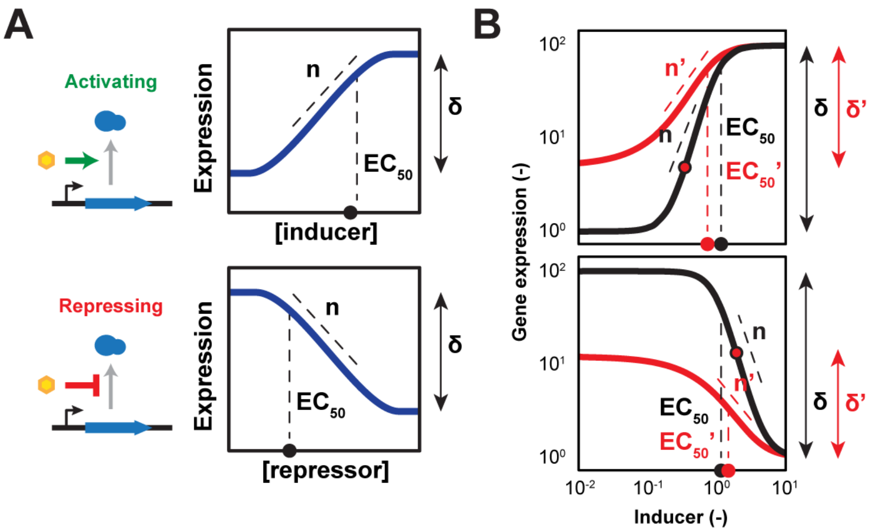

Supplementary Figure S1. Performance metrics based on residual inducer. (**A**) Parameters in the Hill equation. The plot reflects log axes. The dynamic range (δ) is the ratio of maximal over minimal expression levels. The Hill coefficient (n) reflects the slope of the response curve. The half-maximal inducer concentration (EC_{50}) is the inducer concentration that yields the average of the maximal and minimal expression levels. (**B**) Illustration of the impact of residual inducer on the parameter values. Residual inducer stretches the response curve (red) to the left, influencing the value of the apparent dynamic range (δ'), apparent Hill coefficient (n'), and the apparent half-maximal inducer concentration (EC_{50}'). Representative examples are shown for an inducing system (top) and a repressing system (bottom). See Figure 1 for more information.

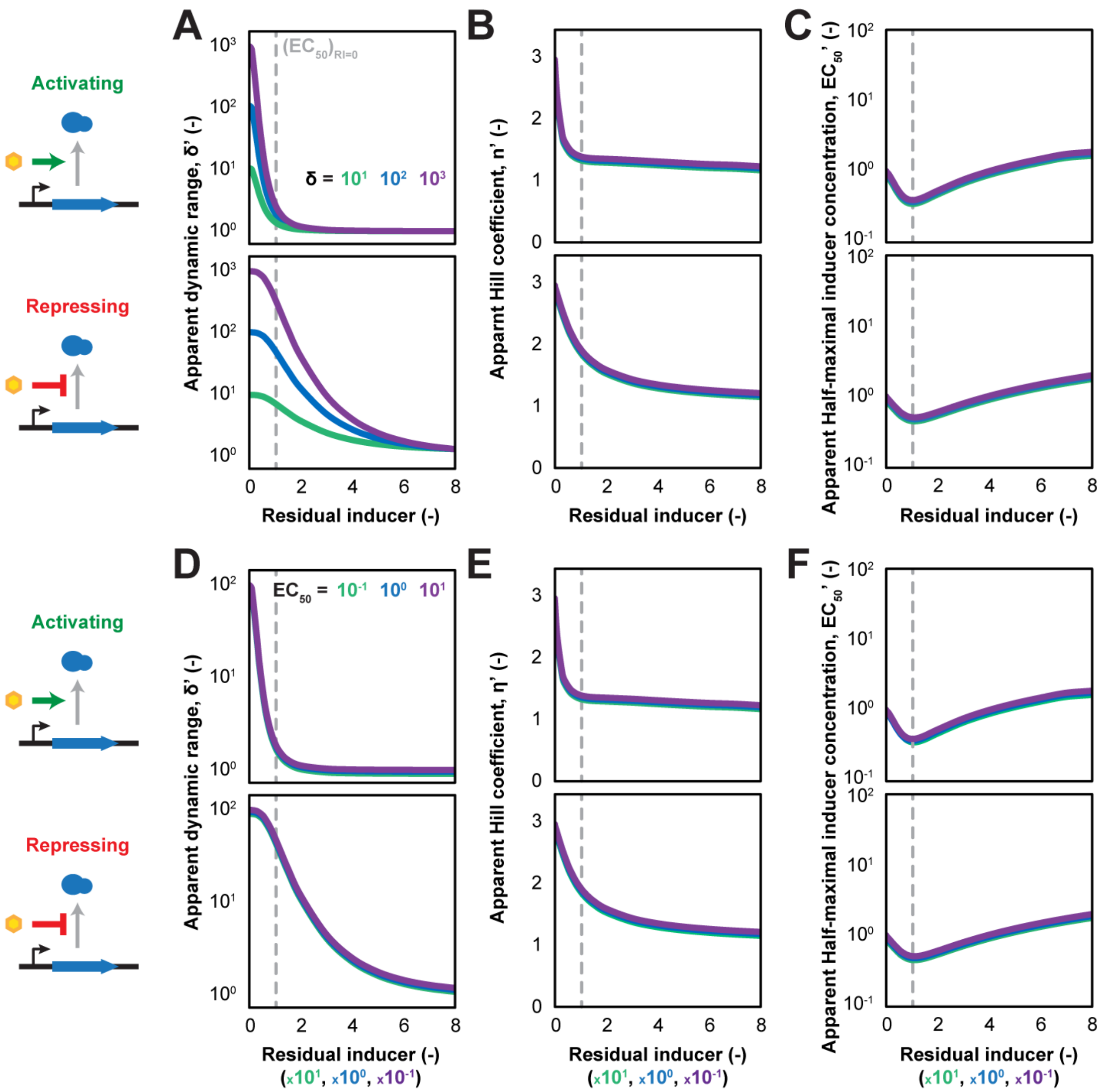

Supplementary Figure S2. Predicted impact of residual inducer for varying values of the dynamic range or the half-maximal inducer concentration. Simulations were conducted similar to those shown in Figure 1. Impact of varying the original dynamic range (δ) on the (**A**) apparent dynamic range (δ'), (**B**) apparent Hill coefficient (n'), and (**C**) apparent half-maximal inducer concentration (EC_{50}'). n = 3, EC_{50} = 1. Top: activating system, Bottom: repressing system. Overlapping curves are slightly offset to avoid masking the other curves. Impact of varying the original half-maximal inducer concentration (EC_{50}) on the (**D**) apparent dynamic range (δ'), (**E**) apparent Hill coefficient (n'), and (**F**) apparent half-maximal inducer concentration (EC_{50}'). The displayed amount of residual inducer was scaled with EC_{50}. Top: activating system, Bottom: repressing system. Overlapping curves are slightly offset to avoid masking the other curves. δ = 100, EC_{50} = 1.

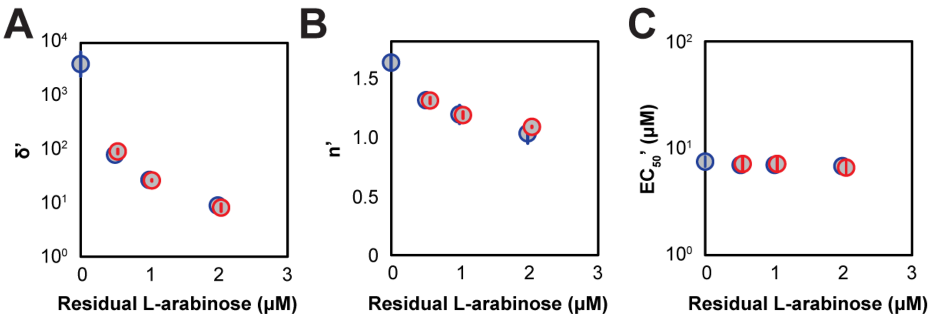

Supplementary Figure S3. Impact of pre-incubating residual L-arabinose on the response parameters. *E. coli* P_{con}-*araE* Δ*araFGH* Δ*araBAD* cells harboring the pUA66-ParaB reporter plasmid were exposed to the indicated concentration of residual L-arabinose either in the overnight culture prior to back-dilution (red) or as part of the back-dilution (blue). See Figure 2 for more information. The parameter values for the apparent dynamic range (δ'), apparent Hill coefficient (n'), and the apparent half-maximal inducer concentration (EC_{50}') were extrapolated by fitting the experimental data to the Hill equation. Values represent the mean and S.E.M. of independent experiments starting with at least three separate colonies. The red circles are slightly offset to the right to avoid concealing the blue circles.

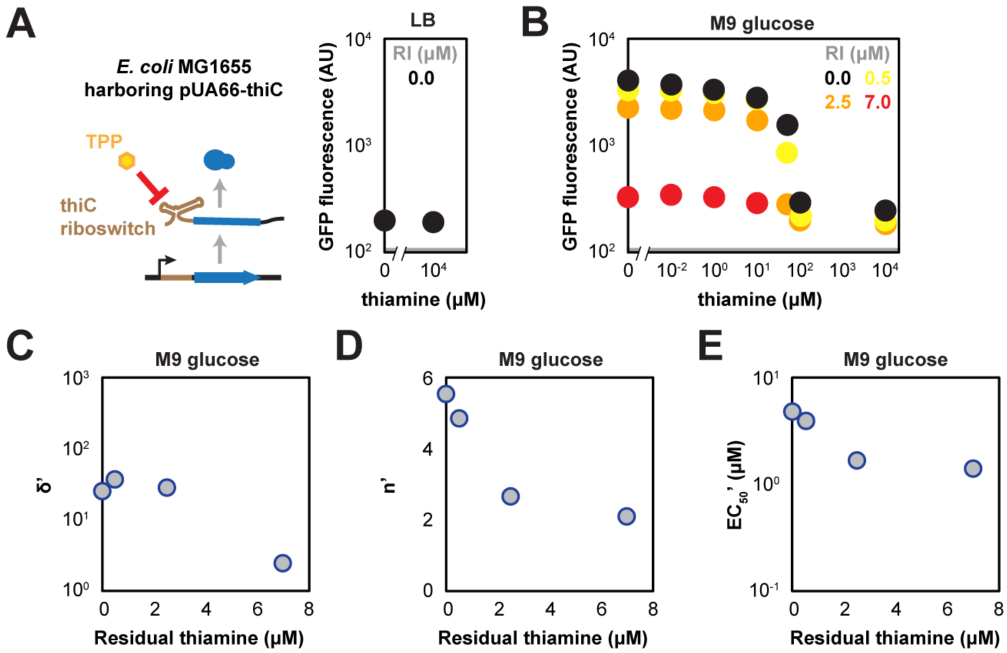

Supplementary Figure S4. Impact of residual thiamine under different growth conditions. *E. coli* cells harboring the pUA66-ThiC reporter plasmid were exposed to the indicated concentration of residual thiamine when grown in (A) LB medium (LB) or (B-E) M9 minimal medium supplemented with 0.4% glucose but no casamino acids (M9 glucose). The parameter values for the apparent dynamic range (δ'), apparent Hill coefficient (n'), and the apparent half-maximal inducer concentration (EC_{50}') were extrapolated by fitting the experimental data to the Hill equation. See Figure 2 for more details.

SUPPLEMENTARY REFERENCES

1. Afroz T, Biliouris K, Kaznessis Y, Beisel CL. Bacterial sugar utilization gives rise to distinct single-cell behaviours. Mol Microbiol. 2014;93: 1093–1103.

2. Afroz T, Biliouris K, Boykin KE, Kaznessis Y, Beisel CL. Trade-offs in engineering sugar utilization pathways for titratable control. ACS Synth Biol. 2015;4: 141–149.
